# Is the proportion of per capita fat supply associated with the prevalence of overweight and obesity? an ecological analysis

**DOI:** 10.1186/s40795-021-00496-2

**Published:** 2022-01-13

**Authors:** Hasinthi Swarnamali, Ranil Jayawardena, Michail Chourdakis, Priyanga Ranasinghe

**Affiliations:** 1 Health and Wellness Unit, Faculty of Medicine, University of Colombo, Colombo, Sri Lanka; 2 Department of Physiology, Faculty of Medicine, University of Colombo, Colombo, Sri Lanka; 3 Institute of Health and Biomedical Innovation, Queensland University of Technology, Brisbane, QLD Australia; 4 Laboratory of Hygiene, Social & Preventive Medicine and Medical Statistics, School of Medicine, Faculty of Health Sciences, Aristotle University of Thessaloniki, University Campus, 54124 Thessaloniki, Greece; 5 Department of Pharmacology, Faculty of Medicine, University of Colombo, Colombo, Sri Lanka

**Keywords:** Ecological analysis, Fat supply, Obesity prevalence, Overweight prevalence

## Abstract

**Background:**

Although it is reported in numerous interventional and observational studies, that a low-fat diet is an effective method to combat overweight and obesity, the relationship at the global population level is not well established. This study aimed to quantify the associations between worldwide per capita fat supply and prevalence of overweight and obesity and further classify this association based on per capita Gross National Income (GNI).

**Methods:**

A total of 93 countries from four GNI groups were selected. Country-specific overweight and obesity prevalence data were retrieved from the most recent WHO Global Health Observatory database. Per capita supply of fat and calories were obtained from the United Nations Food and Agricultural Organization database; FAOSTAT, Food Balance Sheet for years 2014–2016. The categorizations of countries were done based on GNI based classification by the World Bank.

**Results:**

Among the selected countries, the overweight prevalence ranged from 3.9% (India) to 78.8% (Kiribati), while obesity prevalence ranged from 3.6% (Bangladesh) to 46.0% (Kiribati). The highest and the lowest per capita fat supply from total calorie supply were documented in Australia (41.2%) and Madagascar (10.5%) respectively. A significant strong positive correlation was observed between the prevalence of overweight (*r* = 0.64, *p* < 0.001) and obesity (*r* = 0.59, *p* < 0.001) with per capita fat supply. The lower ends of both trend lines were densely populated by the low- and lower-middle-income countries and the upper ends of both lines were greatly populated by the high-income countries.

**Conclusions:**

Per capita fat supply per country is significantly associated with both prevalence of overweight and obesity.

**Supplementary Information:**

The online version contains supplementary material available at 10.1186/s40795-021-00496-2.

## Background

The global prevalence of overweight and obesity has increased in both children and adults during the past 20 years [1]. Overweight and Obesity are the medical conditions in which excess body fat has accumulated to an extent that it may have important public health problems associated with an increased risk of type-2 diabetes mellitus, dyslipidemia, hypertension, and cardiovascular disease (CVD) [2]. The World Health Organization (WHO) describes overweight and obesity as among the most visible, yet neglected public health problems [3]. They threaten public health in both developed and developing countries. Parameters that contribute to variations in the development and consequences of overweight and obesity have been related to multiple risk factors, but the recent epidemic is mainly due to the changes in lifestyle, namely the lack of physical activity and changes in dietary habits [4, 5]. Chronic overfeeding is one of the fundamental risk factors that have been identified, as it accumulates energy stores and leading to the development of overweight and obesity [6]. Therefore, a common approach to combat overweight and obesity has been to limit the energy intake.

Sources of energy are macronutrients and alcohol and most foods and beverages contain combinations of carbohydrates, proteins, and fats macronutrients in varying amounts. Dietary fat intake often has been claimed as responsible for the increase in adiposity [7, 8]. However, total energy balance is what matters most and the focus on dietary fat consumption must be seen through its effects on total energy intake (TEI). A significant positive relationship has been found between the amount of energy from fat and the proportion of the population who are overweight in epidemiological studies [9, 10], and in clinical studies between the level of dietary fat and body-weight gain as well as between the reduction in the dietary fat and weight loss [11, 12].

People from different countries have distinctive food consumption patterns due to variations in availability, affordability, and local dietary habits [13]. At the same time, food production modernization and rising income levels in the last decades have made a range of foods easily available and affordable with less seasonal variation in some countries [14, 15]. Although it is widely reported that a low-fat diet is an effective method of weight loss [16–18], its relationship with overweight and obesity trend at the global population level is not well established. Meanwhile, global patterns, distributions, and heterogeneity of consumption of dietary fat have been exhibited [19]. Therefore, it is useful to look at this relationship with nationally representative data. This study explored the associations of per capita fat supply with the worldwide prevalence of overweight and obesity. Furthermore, we aimed to quantify this relationship further based on per capita Gross National Income (GNI) classifications by the World Bank.

## Methods

The correlation between the prevalence of overweight/obesity in the population and per capita fat supply was investigated using income stratification. For this ecological study, country-specific data were obtained. The countries were selected based on the availability of data for all relevant variables chosen for the study.

### Data sources

The WHO Global Health Observatory (WHO-GHO) database was used to acquire the prevalence rates for overweight and obesity in adults for the year 2016 (the most recent dataset version) [20]. Per capita fat and calorie supply were collected from the United Nations (UN) Food and Agricultural Organization database; FAOSTAT Food Balance Sheet (FAOSTAT-FBS) data for years 2014–16 which was backdated to reflect exposure with delayed presentation [21]. The categorization of countries was done based on the world’s economies classified by the World Bank [22]. FAOSTAT-FBS data by country from 2014–16 with WHO-GHO data in 2016 under four income groups are presented in Supplementary Table 1.

### The WHO-GHO data

The WHO-GHO is a WHO project to disseminate global health data, including statistics by country and information on particular diseases and health interventions [20]. The WHO-GHO collects prevalence data on biological risk factors, such as mean BMI, overweight, and obesity, for WHO member countries using defined methodologies [20]. For the most current and updated datasets version, WHO-GHO statistics on estimated prevalence rates of overweight and obesity (percent of the population aged 18 + with BMI 25 and 30 kg/m^2^ respectively) per country were gathered (2016) [20].

### The FAOSTAT-FBS data

The FAOSTAT database disseminates statistical data collected and maintained by the FAO. FAOSTAT data are provided as a time-series in most domains through a FBS [21]. The FBS provides a complete view of a country's food supply structure over a defined time frame. The FAOSTAT-FBS gives yearly statistics per country on the daily supply of total calories (in kcal per day), protein (in gram per day), and fat (in gram per day). The FAOSTAT FBS data were used to derive the daily caloric supply and macronutrients of fats (animal and plant, in gram/capita/day) [21] by the country for the period between 2014–16.

The number of calories from fat was determined using the Atwater energy density method [23]. The mean values for calories and fat per person per day were calculated over a three-year period (2014–16) to represent typical dietary fat exposure, because obesity begins to develop after repeated exposure to dietary risks, and the mean of three years of fat may also reduce random errors during FAO data gathering and computation. The reasoning for this approach is because it has previously been demonstrated that one to three years is a reasonable time frame for estimating a country's obesity prevalence after exposure to dietary risk [24]. The percentage of calories obtained from fat might therefore be computed by dividing the total daily caloric supply by the amount of calories derived from fat.

### The World Bank data

The World Bank defines the world's economies into four income categories: “high”, “upper-middle”, “lower-middle”, and “low”. Official World Bank estimates of the scale of economies are based on per capita GDP translated to current US dollars ($) using the World Bank Atlas technique [25]. These four classifications of countries are determined by a country’s GNI per capita, which can change with economic growth, inflation, exchange rates, and population [26]. For the current financial year 2020, low-income economies are defined as having a GNP per capita of $1,035 or less in 2019; lower-middle-income economies have a GNP per capita between $1,036 and $4,045; upper-middle-income economies have a GNP per capita between $4,046 and $12,535; and high-income economies have a GNP per capita of $12,536 or more [22].

### Data extraction and analysis

All data were extracted and recorded in Microsoft Excel® [version 2013 for Windows] for analysis by one reviewer (HS) using a standardized form, and correctness was confirmed by a second reviewer (PR). Disagreements in the retrieved data were addressed by discussion, with the assistance of a third reviewer where necessary (RJ). The prevalence estimates of overweight and obesity were matched to the year- and country-specific fat intake variable using data from 93 countries. The World Bank dataset was used to classify an income stratum based on GNP. A correlation coefficient was used to investigate the association between per capita fat supply and other dependent variables such as the prevalence of overweight and obesity. The World Bank income categorization was used to categorize countries for correlation analysis. Pearson's correlation coefficients (r) were computed to assess the strength and direction of the correlations between per capita fat supply and overweight and obesity prevalence. Scatter plots were used to visualize the correlation, and the outcome was examined by constructing a regression line. All countries were named by a three-letter country code in line with International Organization for Standardization 3166, which was taken from the United Nations Statistics Divisions' Terminology Bulletin Country Names and Country Codes for Statistical Use [27].

## Results

Data were analysed for a total of 93 countries among the 4 income groups (low-income = 23; lower-middle-income = 26; upper-middle-income = 23; high-income = 21). With regards to overweight and obesity prevalence data, Kiribati showed the highest overweight and obesity prevalence (78.8% and 46.0% respectively), while India showed the lowest overweight prevalence (3.9%) and Bangladesh showed the lowest prevalence for obesity (3.6%). For the period 2014–16 years, the per capita percentage of fat supply ranged from 10.5–41.2% of total caloric supply, whereas the lowest and highest percentages of per capita fat supply were reported for Madagascar and Australia, respectively.

Table 1 summarizes the correlations based on economic status as defined by the GNI for the included 93 countries. A strong positive Pearson's correlation coefficient was observed between both the prevalence of overweight (*r* = 0.64, *p* < 0.001) and obesity (*r* = 0.59, *p* < 0.001) with per capita fat supply. When countries were categorized based on income; significant positive correlations were exhibited for both overweight and obesity prevalence (*r* = 0.42, *p* = 0.03 for both) in the lower-middle-income countries. And, a significant positive correlation was observed only for overweight prevalence (*r* = 0.53, *p* = 0.01) in the high-income countries. However, there was no significant association in low and upper-middle-income countries for both overweight and obesity prevalence.

**Table 1:** Correlation coefficient and coefficient of determination between per capita fat supply and dependent variables of overweight and obesity based on the economic strata classification

Correlation	Overweight			Obesity		
	r	p	R^2^	r	p	R^2^
All countries	0.64	< 0.001	0.41	0.59	< 0.001	0.34
**Income status**						
Low	0.23	0.28	0.05	0.29	0.17	0.09
Lower middle	0.42	0.03	0.17	0.42	0.03	0.18
Upper middle	0.24	0.27	0.06	0.28	0.08	0.08
High	0.53	0.01	0.28	0.38	0.08	0.14

*r* = Pearson’s correlation coefficient, *p* = Significance, *R*^*2*^ = coefficient of determination

### Analysis of all countries

**Fig. 1:**
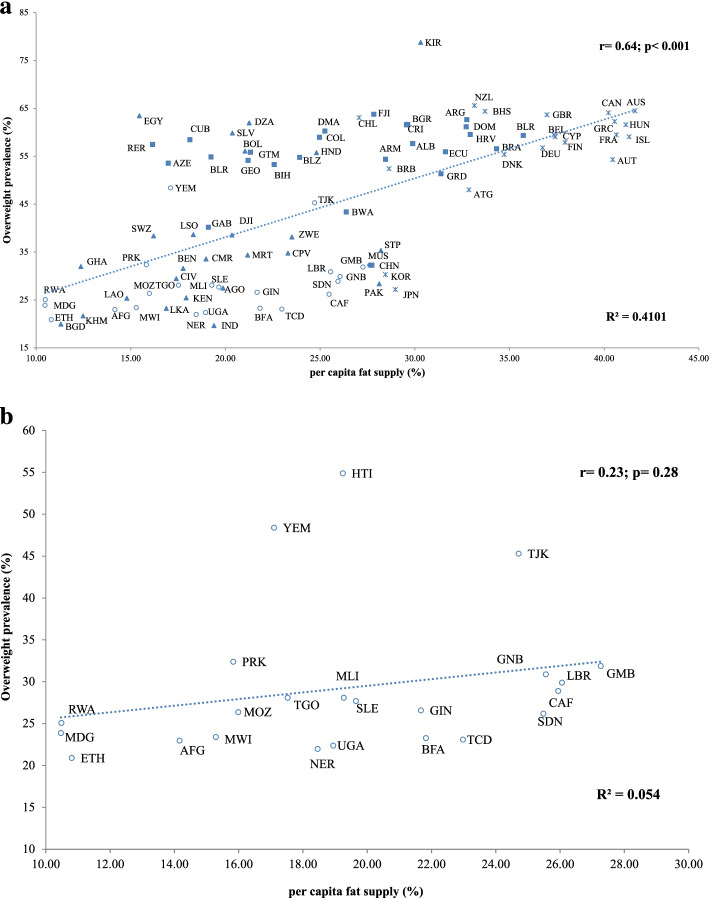
**a** Correlation between per capita fat supply (as a percentage of total calorie supply) and the prevalence of overweight among all selected countries. Afghanistan-AFG; Albania-ALB; Algeria-DZA; Angola- AGO; Antigua and Barbuda-ATG; Argentina-ARG; Armenia-ARM; Australia- AUS; Austria-AUT; Azerbaijan-AZE; Bahamas- BHS; Bangladesh-BGD; Barbados-BRB; Belarus-BLR; Belgium-BEL; Belize-BLZ; Benin-BEN; Bolivia-BOL; Bosnia and Herzegovina-BIH; Botswana-BWA; Brazil-BRA; Bulgaria-BGR; Burkina Faso-BFA; Cape Verde-CPV; Cambodia-KHM; Cameroon-CMR; Canada-CAN; Central African Republic-CAF; Chad-TCD; China-CHN; Chile-CHL; Colombia-COL; Costa Rica- CRI; Cote d'Ivoire-CIV; Croatia-HRV; Cuba-CUB; Cyprus-CYP; Denmark-DNK; Djibouti-DJI; Dominica-DMA; Dominican Republic-DOM; Ecuador-ECU; Egypt-EGY; El Salvador-SLV; Eswatini-SWZ; Ethiopia-ETH; Fiji-FJI; Finland-FIN; France-FRA; Gabon-GAB; Gambia- GMB; Georgia-GEO; Germany-DEU; Ghana-GHA; Greece-GRC; Grenada-GRD; Guatemala-GTM; Guinea-GIN; Guinea Bissau-GNB; Haiti-HTI; Honduras-HND; Hungary-HUN; India-IND; Iceland-ISL; Japan-JPN; Kenya-KEN; Kiribati-KIR; Lao People's Democratic Republic-LAO; Lesotho-LSO; Liberia-LBR; Madagascar-MDG; Malawi-MWI; Mali-MLI; Mauritania-MRT; Mauritius-MUS; Mozambique-MOZ; New Zealand-GBR; Niger-NER; North Korea-NER; Pakistan-PAK; Peru-RER; Rwanda-RWA; Republic of Korea-KOR; Sao Tome and Principe- STP; Sierra Leone-SLE; Sri Lanka-LKA; Sudan-SDN; Tajikistan-TJK; Togo-TGO; Uganda-UGA; United Kingdom-GBR; Yemen-YEM; Zimbabwe- ZWE.  : Low-income;  : Lower-middle-income;  : Upper-middle-income;  : High-income. **b** Correlation between per capita fat supply (as a percentage of total calorie supply) and the prevalence of obesity among all the countries. Afghanistan-AFG; Albania-ALB; Algeria-DZA; Angola- AGO; Antigua and Barbuda-ATG; Argentina-ARG; Armenia-ARM; Australia- AUS; Austria-AUT; Azerbaijan-AZE; Bahamas- BHS; Bangladesh-BGD; Barbados-BRB; Belarus-BLR; Belgium-BEL; Belize-BLZ; Benin-BEN; Bolivia-BOL; Bosnia and Herzegovina-BIH; Botswana-BWA; Brazil-BRA; Bulgaria-BGR; Burkina Faso-BFA; Cape Verde-CPV; Cambodia-KHM; Cameroon-CMR; Canada-CAN; Central African Republic-CAF; Chad-TCD; China-CHN; Chile-CHL; Colombia-COL; Costa Rica- CRI; Cote d'Ivoire-CIV; Croatia-HRV; Cuba-CUB; Cyprus-CYP; Denmark-DNK; Djibouti-DJI; Dominica-DMA; Dominican Republic-DOM; Ecuador-ECU; Egypt-EGY; El Salvador-SLV; Eswatini-SWZ; Ethiopia-ETH; Fiji-FJI; Finland-FIN; France-FRA; Gabon-GAB; Gambia- GMB; Georgia-GEO; Germany-DEU; Ghana-GHA; Greece-GRC; Grenada-GRD; Guatemala-GTM; Guinea-GIN; Guinea-Bissau-GNB; Haiti-HTI; Honduras-HND; Hungary-HUN; India-IND; Iceland-ISL; Japan-JPN; Kenya-KEN; Kiribati-KIR; Lao People's Democratic Republic-LAO; Lesotho-LSO; Liberia-LBR; Madagascar-MDG; Malawi-MWI; Mali-MLI; Mauritania-MRT; Mauritius-MUS; Mozambique-MOZ; New Zealand-GBR; Niger-NER; North Korea-NER; Pakistan-PAK; Peru-RER; Rwanda-RWA; Republic of Korea-KOR; Sao Tome and Principe- STP; Sierra Leone-SLE; Sri Lanka-LKA; Sudan-SDN; Tajikistan-TJK; Togo-TGO; Uganda-UGA; United Kingdom-GBR; Yemen-YEM; Zimbabwe- ZWE.  : Low-income;  : Lower-middle-income;  : Upper-middle-income;  : High-income

The relationship between per capita fat supply and prevalence of both overweight and obesity for all the countries is noted to be logarithmic with strong correlations (Fig. 1a and b respectively). The overweight prevalence of all included countries showed a significant positive correlation (*r* = 0.64, *p* < 0.001) and 41% of the data fit the regression model between per capita fat supply and overweight prevalence (*R*^*2*^ = 0.41) (Fig. 1a). The obesity prevalence of all included countries also showed a significant positive correlation (*r* = 0.59, *p* < 0.001) with the per capita fat supply and 34% of the data fit the regression model between per capita fat supply and obesity prevalence (*R*^*2*^ = 0.34) (Fig. 1b). The regression lines obtained by the correlation analysis indicated an upward tendency, and, as indicated, nearly all included countries scattered around both lines, with only a few outliers (including Egypt and Kiribati). The lower end of these both lines was densely populated by most of the low-income and lower-middle-income countries, except for a few countries as outliers (including Kiribati, Egypt, Algeria, El Salvador, Bolivia). The upper ends of both lines were greatly populated by most of the high-income countries. All upper-middle-income countries (excluding China) scattered around the middle to the upper end of the regression line.

### Analysis based on GNI

**Fig. 2:**
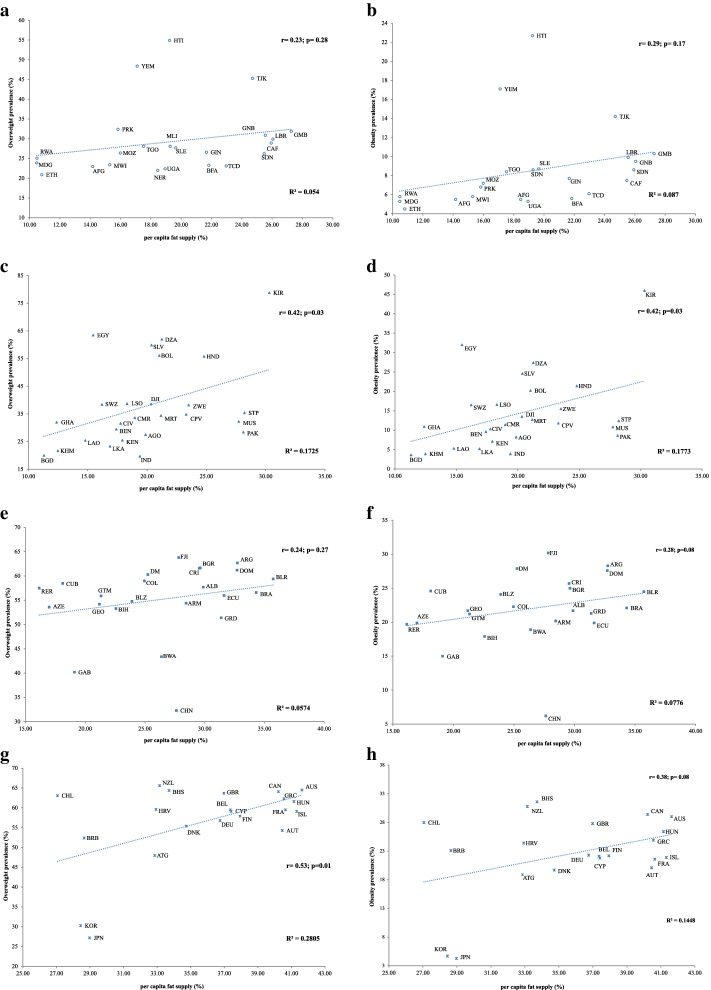
**a** Correlation between per capita fat supply (as a percentage of total calorie supply) and the prevalence of overweight among low-income countries. Afghanistan-AFG; Burkina Faso-BFA; Central African Republic-CAF; Chad-TCD; Ethiopia- ETH; Gambia-GMB; Guinea-GIN; Guinea Bissau-GNB; Haiti-HTI; Liberia-LBR; Madagascar-MDG; Malawi-MWI; Mali-MLI; Mozambique-MOZ; Niger-NER; North Korea-NER; Rwanda-RWA; Sierra Leone-SLE; Sudan-SDN; Tajikistan-TJK; Togo-TGO; Uganda-UGA; Yemen-YEM. **b** Correlation between per capita fat supply (as a percentage of total calorie supply) and prevalence of obesity among low-income countries. Afghanistan-AFG; Burkina Faso-BFA; Central African Republic-CAF; Chad-TCD; Ethiopia- ETH; Gambia-GMB; Guinea-GIN; Guinea Bissau-GNB; Haiti-HTI; Liberia-LBR; Madagascar-MDG; Malawi-MWI; Mali-MLI; Mozambique-MOZ; Niger-NER; North Korea-NER; Rwanda-RWA; Sierra Leone-SLE; Sudan-SDN; Tajikistan-TJK; Togo-TGO; Uganda-UGA; Yemen-YEM. **c** Correlation between per capita fat supply (as a percentage of total calorie supply) and prevalence of overweight among lower-middle-income countries. Algeria-DZA; Angola-AGO; Bangladesh-BGD; Benin-BEN; Bolivia-BOL; Cambodia-KHM; Cameroon-CMR; Cape Verde-CPV; Cote d'Ivoire-CIV; Djibouti-DJI; Egypt-EGY; El Salvador-SLV; Eswatini-SWZ; Ghana-GHA; Honduras-HND; India-IND; Kenya-KEN; Kiribati-KIR; Lao People's Democratic Republic-LAO; Lesotho-LSO; Mauritania-MRT; Mauritius-MUS; Pakistan-PAK; Sao Tome and Principe-STP; Sri Lanka-LKA; Zimbabwe-ZWE. **d** Correlation between per capita fat supply (as a percentage of total calorie supply) and prevalence of obesity among lower-middle-income countries. Algeria-DZA; Angola-AGO; Bangladesh-BGD; Benin-BEN; Bolivia-BOL; Cambodia-KHM; Cameroon-CMR; Cape Verde-CPV; Cote d'Ivoire-CIV; Djibouti-DJI; Egypt-EGY; El Salvador- SLV; Eswatini-SWZ; Ghana-GHA; Honduras-HND; India-IND; Kenya-KEN; Kiribati-KIR; Lao People's Democratic Republic-LAO; Lesotho-LSO; Mauritania-MRT; Mauritius-MUS; Pakistan-PAK; Sao Tome and Principe-STP; Sri Lanka-LKA; Zimbabwe-ZWE. **e** Correlation between per capita fat supply (as a percentage of total calorie supply) and prevalence of overweight among upper-middle-income countries. Albania-ALB; Argentina-ARG; Armenia-ARM; Azerbaijan-AZE; Belarus-BLR; Belize-BLZ; Bosnia and Herzegovina-BIH; Botswana-BWA; Brazil-BRA; Bulgaria-BGR; China-CHN; Colombia-COL; Costa Rica-CRI; Cuba-CUB; Dominica-DM; Dominican Republic-DOM; Ecuador-ECU; Fiji- FJI; Gabon-GAB; Georgia-GEO; Grenada-GRD; Guatemala-GTM; Peru-RER. **f** Correlation between per capita fat supply (as a percentage of total calorie supply) and prevalence of obesity among upper-middle-income countries. Albania-ALB; Argentina-ARG; Armenia-ARM; Azerbaijan-AZE; Belarus-BLR; Belize- BLZ; Bosnia and Herzegovina- BIH; Botswana- BWA; Brazil- BRA; Bulgaria- BGR; China- CHN; Colombia- COL; Costa Rica- CRI; Cuba- CUB; Dominica- DM; Dominican Republic- DOM; Ecuador- ECU; Fiji- FJI; Gabon- GAB; Georgia- GEO; Grenada- GRD; Guatemala- GTM; Peru- RER. **g** Correlation between per capita fat supply (as a percentage of total calorie supply) and prevalence of overweight among high-income countries. Antigua and Barbuda- ATG; Australia- AUS; Austria- AUT; Bahamas- BHS; Barbados- BRB; Belgium- BEL; Canada- CAN; Chile- CHL; Croatia- HRV; Cyprus- CYP; Denmark- DNK; Finland- FIN; France- FRA; Germany- DEU; Greece- GRC; Hungary- HUN; Iceland- ISL; Japan- JPN; New Zealand- GBR; Republic of Korea- KOR; United Kingdom- GBR. **h** Correlation between per capita fat supply (as a percentage of total calorie supply) and prevalence of obesity among high-income countries. Antigua and Barbuda- ATG; Australia- AUS; Austria- AUT; Bahamas- BHS; Barbados- BRB; Belgium- BEL; Canada- CAN; Chile- CHL; Croatia- HRV; Cyprus- CYP; Denmark- DNK; Finland- FIN; France- FRA; Germany- DEU; Greece- GRC; Hungary- HUN; Iceland- ISL; Japan- JPN; New Zealand- GBR; Republic of Korea- KOR; United Kingdom- GBR

Scatter plots depicting the association between the above variables in each income group based on GNP were also generated (Fig. 2a-h). All of the regression lines produced by the correlation analysis similarly indicated a upward trend.

### Analysis of low-income countries

Among the low-income countries, Haiti showed the highest prevalence of both overweight (54.9%) and obesity (22.7%) while Ethiopia showed the lowest prevalence of both overweight (20.9%) and obesity (4.5%). However, Gambia and Madagascar showed the highest (27.3%) and lowest (10.5%) per capita fat supply among the low-income group respectively. Both overweight and obesity prevalence were not significantly correlated with per capita fat supply (*r* = 0.23, *p* = 0.28 and *r* = 0.29, *p* = 0.17 correspondingly) (Fig. 2a and 2b). However, the correlation effect significantly changed after removing the outliers (Yemen, Haiti) from the analysis, which then gave a significant correlation at both overweight (*r* = 0.49, *p* = 0.02) and obesity (*r* = 0.67, *p* < 0.001) prevalence with 24% and 45% of variations for overweight (*R*^*2*^ = 0.24) and obesity (*R*^*2*^ = 0.45) respectively (Supplementary Table 2).

### Analysis of lower-middle-income countries

The prevalence of overweight ranged from 19.7% (India) to 78.8% (Kiribati), while obesity ranged from 3.6% (Bangladesh) to 46.0% (Kiribati). A wide range of per capita fat supply, representing 11.3% Bangladesh and 30.3% Kiribati was found in this group. It is noteworthy that Kiribati had the highest prevalence of overweight and obesity, as well as the highest per capita fat supply. In this income category, both overweight and obesity prevalence were significantly correlated with per capita fat supply (*r* = 0.42, *p* = 0.03 and *r* = 0.42, *p* = 0.03 respectively) with the variation of 17% for overweight (*R*^*2*^ = 0.17) and 18% for obesity (*R*^*2*^ = 0.18) (Fig. 2c and d).

### Analysis of upper-middle-income countries

Fiji had the highest prevalence of both overweight (30.2%) and obesity (63.8%), while China was the lowest country for those values (32.3% and 6.2% respectively). Per capita fat supply was ranged from 16.2% (Peru) to 35.7% (Belarus) among the upper-middle-income group. Countries in the upper-middle-income group did not show a significant correlation between per capita fat supply and prevalence of both overweight and obesity (*r* = 24, *p* = 0.27, and *r* = 0.28, *p* = 0.08 respectively) (Fig. 2e and 2f). However, after removing one outlier (China), a significant correlation was reported with obesity prevalence (*r* = 0.43, *p* = 0.04) with 18% of the variation (*R*^*2*^ = 0.18) (Supplementary Table 2). All upper-middle-income countries except a couple of countries such as Georgia and China were clustered close to the regression line.

### Analysis of high-income countries

Among high-income countries, Japan showed the lowest prevalence for both overweight (27.2%) and obesity (4.3%). New Zealand presented the highest overweight prevalence (65.6%) whereas the Bahamas showed the highest obesity prevalence (31.6%). The per capita fat supply ranged from 27.1% (Chile) to 41.6% (Australia). Only overweight prevalence significantly correlated with per capita fat supply (*r* = 0.53, *p* = 0.01) with 28% of the variation (*R*^*2*^ = 0.28) (Fig. 2g). However, per capita fat supply did not significantly correlate with the obesity prevalence (*r* = 0.38, *p* = 0.08) in the high-income group (Fig. 2h). Almost all included countries in the regression line generated from the correlation analysis among the high-income group scattered around the line, with two countries, (Republic of Korea and Japan) as outliers.

## Discussion

The results of our analysis have demonstrated that the per capita fat supply is a very good predictor for the prevalence of overweight and obesity at the country level. The link was found to be linear, with a substantial association between per capita fat supply and the incidence of both overweight and obesity. The correlation we have found in this study between fat intake and overweight and obesity is compatible with that demonstrated in epidemiological studies [28–30], and in clinical studies [31, 32] which also shows the positive relationship between dietary fat consumption and increase of body-weight. Furthermore, in the pooled analysis, our findings on the link between fat consumption and the dependent variables of overweight and obesity demonstrate a substantial positive correlation.

However, the pattern of association of per capita fat supply to overweight and obesity differs according to income strata. According to that, a significant correlation between per capita fat supply and variables of both overweight and obesity was noted in the lower-middle-income group. At the same time, a significant correlation was also noted in high-income strata as well, but only for the overweight prevalence. Though the correlations were not significant in other sub-categories, that effect significantly changed for several sub-groups after removing few outliers. For example, the correlation coefficient was significantly noted for both variables of overweight and obesity in the low-income group after removing data from Yemen and Haiti, which were considered outliers. And, linear regression models between the per capita fat supply and prevalence of both overweight and obesity also increased after removing those two outliers. Moreover, correlation changed as significant for obesity in the upper-middle-income group after removing one outlier (China). The lack of significant correlation in the remaining two sub-categories (overweight in upper-middle-income and obesity in high-income groups) may have been due to the insufficient data points, with a smaller number of countries.

The joint WHO/FAO consultation on fats and oils proposed that dietary fat should supply a minimum of 15% of TEI, but not exceed 30–35% of TEI for most adults [33]. The country-specific analysis of the current study has found a range of 10.5–41.6% of fat energy ratio between 2014–16. According to our analysis, seven countries fell below the minimum recommendation of 15% of dietary energy supply from fat, all of which were in the low-income (Ethiopia, Ghana, Cambodia, and Lao People’s Democratic Republic) and lower-middle-income (Madagascar, Rwanda, and Afghanistan) categories. Thirteen countries exceeded the 35% maximum, with twelve countries being in the high-income group and one in the upper-middle-income group. It appears that the countries with an excess of per capita fat supply are generally economically developed countries.

When analyzing the factors that influence fat consumption patterns, assessments of fat consumption statistics suggest that persons in the lowest socioeconomic level in most developed countries take greater fatty foods [34]. Studies have shown that gender [35] and age [36] differences were also found in consumption of fatty foods. Moreover, urbanization is also strongly associated with the increasing consumption of fat in developing countries [37]. In addition to that, the physical environment, level of education, sociological, and individual factors also affect the altitude of fat consumption. Therefore, this entire phenomenon is part of an overall change in food habits and then determines the total quantity of fat availability at the country level.

Obesity caused by a high-fat diet is explained by a number of physiological processes. These include low satiating effects, as well as changes in hormones involved in energy balance [38]. More dietary fat leads to higher obesity because fat contains 9 kcal/g of energy compared to 4 kcal/g for carbs and protein [39]. It is evident that high-fat meals have a high energy density, and so the overall fat content of the diet is an important determinant in energy balancing. Furthermore, weaker satiety signals from fats than from carbohydrate and protein have been proposed to involved in fat-rich diet overconsumption of calories [40]. The extra eating caused by fat-rich diets is due to their post-ingestion effect, which may increase food intake by conditioning sensory preference [41]. Furthermore, protein and carbohydrate stimulate significant auto-regulatory modifications in oxidation in response to variations in intake, but fat is at the bottom of an oxidative hierarchy that controls fuel choices [42].

Due to unavailability from relevant UN organizations, all information on the two variables we utilized in our research was not equally available for all nations throughout the world. As a result, the number of countries used in this analysis was limited to those possessing relevant data. FAO, WHO, and the World Bank are international institutions that provide specialist information in their respective areas. Before they were released, they analyzed these data in terms of their potential uses, such as scientific research and decision making. This indicates that while mistakes have been decreased, certain inaccuracies relating to reporting quality may still exist in the data.

### Limitations

It must be noted that there are several limitations to this study. Firstly, there may be some possible confounding variables (e.g., prevalence of physical inactivity, absolute total calories, and fat consumption or food wastage) that were not included in our study and may have influenced the association we discovered. However, it is impossible to determine what such factors may be in the current investigation. Second, we could only utilize an international food database that measures per capita calorie and fat supply, not actual human consumption. However, there are no direct assessments of actual human intake that can account for food waste and give exact statistics of food consumption globally. Third, because the data studied are computed per capita in each country, we could only find the correlation at the country level, which does not always equate to the same associations at the individual level. Fourth, BMI values of 25 kg/m2^2^ and 30 kg/m^2^ were used as the cut-off points for classifying overweight and obesity in this study cohort. However, different cut-off points can be employed to define overweight and obesity among different ethnic groups. We used WHO country reports for this study to possibly reduce substantial discrepancies between countries, although more recently published data on the prevalence of overweight and obesity are available for some countries. Finally, the findings cannot be extended to the individual level because this is an ecological study.

### Future perspectives

Prospective cohort studies and intervention studies are recommended in each country to investigate this link further. Furthermore, assessing heterogeneity in various amounts of animal fat and plant fat is important for determining a true depiction of the connection at the national level. Country-specific nutrition education messages that warn consumers about the consequences of a high-fat diet and how to restrict sources of fat consumption to maintain a healthy body weight are critical. Relevant authorities in the countries should implement food regulations, active initiatives to raise awareness of the consequences of high fat consumption and its sources, and related taxes on food industry based on the amount of fat used as an ingredient so that the public would make rational decisions.

## Conclusion

In all nations, significant positive associations were found between the prevalence of overweight and obesity and country-specific per capita fat supply. The regression lines derived by the correlation analysis indicated an increasing trend. Most low-income and lower-middle-income nations were densely populated at the lower ends of both lines, indicating a low incidence of both overweight and obesity and a per capita low-fat supply. In contrast, most high-income nations filled the higher ends of both lines, showing a high incidence of both overweight and obesity with a per capita high fat supply.

## Supplementary Information

**Additional file 1.** Detailed information on the country-level prevalence of overweight and obesity and per capita fat supply and total calories

**Additional file 2.** Correlation coefficient and coefficient of determination between per capita fat supply and dependent variables-overweight and obesity in two income groups after removing outliers

## Data Availability

All data for this study are publicly available and are ready for the public to download at no cost from the official websites of the World Bank [22], the WHO [20], and FAO [21]. Usage of these data for this research falls within the UN agency’s public permission in their terms and conditions. There is no need to have formal permission to use the data for this study as public access to the databases is open. The sources and data robustness has been described in the section of “Methods”. Furthermore, detailed information on the country-level prevalence of overweight and obesity and per capita fat supply and total calories are contained within Supplementary Table 1.

## Abbreviations

BMI: Body Mass Index
CVD: Cardiovascular Disease
FAO: Food and Agricultural Organization
FBS: Food Balance Sheet
GHO: Global Health Observatory
GNI: Gross National Income
MUFA: Monounsaturated Fatty Acid
PUFA: Polyunsaturated Fatty Acid
SFA: Saturated Fatty Acid
TEI: Total energy intake
TG: Triglyceride
UN: United Nations
WHO: World Health Organization
