# Supplementary material for: Is the proportion of per capita fat supply associated with the prevalence of overweight and obesity? an ecological analysis
Source: BMC Nutr. 2022 Jan 13;8:4. doi: 10.1186/s40795-021-00496-2 (PMC8756625; doi:10.1186/s40795-021-00496-2)
Supplement: Supplementary file 1 — Additional file 1. Detailed information on the country-level prevalence of overweight and obesity and per capita fat supply and total calories [file 40795_2021_496_MOESM1_ESM.docx]

| **Country**  **Supplementary Table 1:** Detailed information on the country-level prevalence of overweight and obesity and per capita fat supply and total calories | **Prevalence data of WHO(GHO) database (2016)** | | | **FAOSTAT Food Balance Sheet data** | | | | | | |
| --- | --- | --- | --- | --- | --- | --- | --- | --- | --- | --- |
|  |  |  |  | **2014** | | **2015** | | | **2016** | |
|  |  |  |  | **Total calorie supply**  **(kcal/capita/day)** | **Fat supply**  **(g/capita/**  **day)** | **Total calorie supply**  **(kcal/capita/day)** | | **Fat supply**  **(g/capita/**  **day)** | **Total calorie supply**  **(kcal/capita/day)** | **Fat supply**  **(g/capita/**  **day)** |
|  | **Obesity (%)** | **Overweight (%)** | |  |  |  |  |  |  |  |
| **Low-income economies** | | | | |  |  | |  |  |  |
| Afghanistan | 5.5 | | 23.0 | 2019 | 32.8 | 1978 | | 30.4 | 2001 | 31.2 |
| Burkina Faso | 5.6 | | 23.3 | 2708 | 63.6 | 2713 | | 66.1 | 2726 | 67.9 |
| Central African Republic | 7.5 | | 26.2 | 1820 | 51.7 | 1758 | | 50.0 | 1755 | 49.3 |
| Chad | 6.1 | | 23.1 | 2112 | 48.8 | 2131 | | 56.9 | 2133 | 57.2 |
| Ethiopia | 4.5 | | 20.9 | 2234 | 26.5 | 2254 | | 27.5 | 2296 | 27.4 |
| Gambia | 10.3 | | 31.9 | 2581 | 77.9 | 2540 | | 77.3 | 2539 | 76.9 |
| Guinea | 7.7 | | 26.6 | 2810 | 66.9 | 2807 | | 68.8 | 2816 | 67.4 |
| Guinea-Bissau | 9.5 | | 29.9 | 2149 | 63.5 | 2112 | | 61.9 | 2176 | 60.9 |
| Haiti (HT) | 22.7 | | 54.9 | 2143 | 46.6 | 2145 | | 45.2 | 2135 | 45.5 |
| Korea, Dem. People's Rep. | 6.8 | | 32.4 | 2080 | 37.2 | 2093 | | 37.8 | 2058 | 34.7 |
| Liberia | 9.9 | | 30.9 | 2158 | 61.5 | 2122 | | 62.5 | 2123 | 57.9 |
| Madagascar | 5.3 | | 23.9 | 1992 | 22.5 | 1928 | | 22.5 | 1925 | 23.0 |
| Malawi | 5.8 | | 23.4 | 2612 | 42.9 | 2596 | | 43.8 | 2554 | 45.1 |
| Mali | 8.6 | | 28.1 | 2833 | 62.4 | 2820 | | 61.0 | 2897 | 59.7 |
| Mozambique | 7.2 | | 26.4 | 2310 | 41.2 | 2339 | | 42.3 | 2362 | 41.0 |
| Niger | 5.5 | | 22.0 | 2546 | 52.5 | 2555 | | 52.2 | 2577 | 52.8 |
| Rwanda | 5.8 | | 25.1 | 2268 | 24.5 | 2242 | | 26.4 | 2252 | 27.8 |
| Sierra Leone | 8.7 | | 27.7 | 2475 | 54.7 | 2447 | | 54.0 | 2450 | 52.2 |
| Sudan | 8.6 | | 28.9 | 2438 | 71.5 | 2438 | | 70.2 | 2441 | 69.1 |
| Tajikistan | 14.2 | | 45.3 | 2093 | 57.7 | 2095 | | 56.9 | 2106 | 58.2 |
| Togo | 8.4 | | 28.1 | 2431 | 40.4 | 2464 | | 59.2 | 2379 | 42.28 |
| Uganda | 5.3 | | 22.4 | 2259 | 49.1 | 2179 | | 45.0 | 2132 | 44.3 |
| Yemen | 17.1 | | 48.4 | 2137 | 40.2 | 2142 | | 40.8 | 1987 | 38.0 |
| **Lower-middle-income economies** | | | | |  | | |  |  |  |
| Algeria | 27.4 | | 62.0 | 3374 | 79.2 | | 3371 | 78.9 | 3359 | 80.5 |
| Angola | 8.2 | | 27.5 | 2246 | 52.4 | | 2191 | 47.9 | 2188 | 46.0 |
| Bangladesh | 3.6 | | 20.0 | 2536 | 30.9 | | 2553 | 32.3 | 2573 | 33.0 |
| Benin | 9.6 | | 29.5 | 2735 | 52.1 | | 2720 | 52.3 | 2734 | 54.0 |
| Bolivia | 20.2 | | 56.1 | 2277 | 51.7 | | 2329 | 55.2 | 2335 | 55.3 |
| Cabo Verde | 11.8 | | 34.8 | 2499 | 65.3 | | 2502 | 64.8 | 2514 | 64.4 |
| Cambodia | 3.9 | | 21.7 | 2463 | 34.6 | | 2458 | 34.0 | 2458 | 33.7 |
| Cameroon | 11.4 | | 33.6 | 2638 | 55.4 | | 2666 | 56.4 | 2685 | 56.6 |
| Cote d'Ivoire | 10.3 | | 31.6 | 2548 | 49.8 | | 2598 | 51.9 | 2652 | 52.3 |
| Djibouti | 13.5 | | 38.6 | 2635 | 59.5 | | 2710 | 61.6 | 2665 | 59.9 |
| Egypt | 32.0 | | 63.5 | 3378 | 57.3 | | 3365 | 58.0 | 3342 | 57.9 |
| El Salvador | 24.6 | | 59.9 | 2585 | 58.3 | | 2585 | 59.3 | 2620 | 58.7 |
| Eswatini | 16.5 | | 38.4 | 2379 | 45.0 | | 2291 | 40.5 | 2388 | 41.7 |
| Ghana | 10.9 | | 32.0 | 2979 | 40.8 | | 2921 | 39.6 | 2925 | 40.9 |
| Honduras | 21.4 | | 55.8 | 2535 | 70.8 | | 2560 | 70.4 | 2591 | 70.6 |
| India | 3.9 | | 19.7 | 2442 | 52.4 | | 2461 | 52.6 | 2496 | 54.4 |
| Kenya | 7.1 | | 25.5 | 2154 | 42.0 | | 2172 | 42.9 | 2120 | 43.5 |
| Kiribati | 46.0 | | 78.8 | 3072 | 103.9 | | 3104 | 104.7 | 3080 | 103.1 |
| Lao People's Dem. Republic | 5.3 | | 25.4 | 2759 | 47.3 | | 2942 | 47.9 | 3080 | 49.0 |
| Lesotho | 16.6 | | 38.7 | 2318 | 47.7 | | 2134 | 43.1 | 1846 | 37.4 |
| Mauritania | 12.7 | | 34.4 | 2924 | 72.7 | | 2835 | 65.5 | 2749 | 62.2 |
| Mauritius | 10.8 | | 32.3 | 3003 | 92.9 | | 3066 | 92.6 | 2986 | 93.5 |
| Pakistan | 8.6 | | 28.4 | 2316 | 71.3 | | 2318 | 73.2 | 2314 | 72.5 |
| Sao Tome and Principe | 12.4 | | 35.4 | 2327 | 74.0 | | 2324 | 72.5 | 2413 | 74.9 |
| Sri Lanka | 5.2 | | 23.3 | 2582 | 48.1 | | 2589 | 49.1 | 2585 | 48.2 |
| Zimbabwe | 15.5 | | 38.2 | 2199 | 57.1 | | 2172 | 56.6 | 2176 | 57.3 |
| **Upper-middle-income economies** | | | | |  | | |  | |  |
| Albania | 21.7 | | 57.7 | 3274 | 108.8 | | 3258 | 107.9 | 3352 | 111.4 |
| Argentina | 28.3 | | 62.7 | 3237 | 116.5 | | 3276 | 118.5 | 3219 | 119.0 |
| Armenia | 20.2 | | 54.4 | 3077 | 97.9 | | 3080 | 96.4 | 3059 | 97.0 |
| Azerbaijan | 19.9 | | 53.6 | 3073 | 57.6 | | 3074 | 58.3 | 3085 | 58.4 |
| Belarus | 24.5 | | 59.4 | 3375 | 136.2 | | 3206 | 127.8 | 3295 | 128.1 |
| Belize | 24.1 | | 54.8 | 2655 | 69.6 | | 2662 | 70.8 | 2683 | 72.3 |
| Bosnia and Herzegovina | 17.9 | | 53.3 | 3196 | 79.8 | | 3255 | 81.2 | 3299 | 83.6 |
| Botswana | 18.9 | | 43.4 | 2404 | 71.7 | | 2386 | 69.0 | 2370 | 69.1 |
| Brazil | 22.1 | | 56.6 | 3301 | 124.0 | | 3238 | 122.9 | 3236 | 125.7 |
| Bulgaria | 25.0 | | 61.7 | 2866 | 90.3 | | 2753 | 93.6 | 2846 | 94.6 |
| China | 6.2 | | 32.3 | 3146 | 96.8 | | 3184 | 97.9 | 3169 | 96.8 |
| Colombia | 22.3 | | 59.0 | 2949 | 84.6 | | 2989 | 82.4 | 3046 | 82.1 |
| Costa Rica | 25.7 | | 61.6 | 2902 | 93.3 | | 2917 | 96.0 | 2994 | 100.2 |
| Cuba | 24.6 | | 58.5 | 3357 | 67.0 | | 3319 | 66.0 | 3409 | 70.1 |
| Dominica | 27.9 | | 60.3 | 2958 | 82.0 | | 2996 | 84.4 | 2965 | 83.8 |
| Dominican Republic | 27.6 | | 61.2 | 2768 | 98.6 | | 2740 | 100.3 | 2835 | 104.3 |
| Ecuador | 19.9 | | 56.0 | 2603 | 94.9 | | 2599 | 90.3 | 2577 | 88.1 |
| Fiji | 30.2 | | 63.8 | 2900 | 87.9 | | 2898 | 87.9 | 2916 | 93.7 |
| Gabon | 15.0 | | 40.2 | 2753 | 58.1 | | 2749 | 59.1 | 2688 | 56.5 |
| Georgia | 21.7 | | 54.2 | 2926 | 67.7 | | 2918 | 69.1 | 2944 | 70.1 |
| Grenada | 21.3 | | 51.4 | 2372 | 82.1 | | 2415 | 86.6 | 2383 | 81.4 |
| Guatemala | 21.2 | | 55.9 | 2449 | 57.3 | | 2504 | 60.0 | 2517 | 59.8 |
| Peru | 19.7 | | 57.5 | 2737 | 49.1 | | 2783 | 49.4 | 2743 | 49.8 |
| **High-income economies** | | | | |  | | | | |  |
| Antigua and Barbuda | 18.9 | | 48.0 | 2411 | 87.7 | | 2419 | 89.1 | 2387 | 76.9 |
| Australia | 29.0 | | 64.5 | 3316 | 152.8 | | 3292 | 152.5 | 3316 | 108.1 |
| Austria | 20.1 | | 54.3 | 3738 | 167.1 | | 3715 | 167.6 | 3687 | 108.4 |
| Bahamas | 31.6 | | 64.4 | 2117 | 82.1 | | 2016 | 76.0 | 2062 | 62.6 |
| Barbados | 23.1 | | 52.4 | 2906 | 91.0 | | 2932 | 94.1 | 2926 | 90.4 |
| Belgium | 22.1 | | 59.5 | 3749 | 156.6 | | 3845 | 161.5 | 3759 | 100.4 |
| Canada | 29.4 | | 64.1 | 3450 | 153.9 | | 3438 | 156.7 | 3489 | 100.8 |
| Chile | 28.0 | | 63.1 | 3008 | 91.4 | | 2982 | 90.2 | 3000 | 88.1 |
| Croatia | 24.4 | | 59.6 | 2968 | 107.8 | | 3033 | 111.2 | 3120 | 90.0 |
| Cyprus | 21.8 | | 59.1 | 2621 | 111.2 | | 2601 | 106.0 | 2599 | 79.1 |
| Denmark | 19.7 | | 55.4 | 3340 | 127.9 | | 3315 | 126.8 | 3335 | 111.6 |
| Finland | 22.2 | | 57.9 | 3329 | 140.6 | | 3317 | 139.9 | 3334 | 116.9 |
| France | 21.6 | | 59.5 | 3509 | 159.3 | | 3524 | 158.7 | 3537 | 111.9 |
| French Polynesia | 22.3 | | 56.8 | 2996 | 128.9 | | 2957 | 128.6 | 2931 | 97.4 |
| Germany | 24.9 | | 62.3 | 3541 | 143.4 | | 3551 | 143.7 | 3574 | 105.0 |
| Greece | 26.4 | | 61.6 | 3431 | 154.4 | | 3383 | 151.0 | 3331 | 103.6 |
| Hungary | 21.9 | | 59.1 | 3118 | 138.5 | | 3223 | 148.4 | 3260 | 87.1 |
| Iceland | 4.3 | | 27.2 | 3465 | 157.8 | | 3548 | 164.0 | 3574 | 139.3 |
| Japan | 30.8 | | 65.6 | 2717 | 86.6 | | 2697 | 87.0 | 2696 | 85.5 |
| New Caledonia | 4.7 | | 30.3 | 2816 | 118.6 | | 2780 | 120.4 | 2739 | 82.0 |
| New Zealand | 27.8 | | 63.7 | 3153 | 115.9 | | 3151 | 115.8 | 3148 | 91.0 |
| Republic of Korea | 18.9 | | 48.0 | 3332 | 104.5 | | 3340 | 106.3 | 3335 | 95.9 |
| United Kingdom | 29.0 | | 64.5 | 3414 | 140.6 | | 3410 | 140.7 | 3409 | 104.7 |

1. The prevalence for overweight and obesity; from WHO Global Health Observatory (GHO) database for the year 2016
2. Per capita fat and calorie supply; from United Nations (UN) Food and Agricultural Organization database; FAOSTAT, Food Balance Sheet data for the year 2014-2016
3. Categorization of countries; based on the world’s economies classified by the World Bank
