# Supplementary material for: Is the proportion of per capita fat supply associated with the prevalence of overweight and obesity? an ecological analysis
Source: BMC Nutr. 2022 Jan 13;8:4. doi: 10.1186/s40795-021-00496-2 (PMC8756625; doi:10.1186/s40795-021-00496-2)
Supplement: Supplementary file 2 — Additional file 2. Correlation coefficient and coefficient of determination between per capita fat supply and dependent variables-overweight and obesity in two income groups after removing outliers [file 40795_2021_496_MOESM2_ESM.docx]

**Supplementary Table 2:** Correlation coefficient and coefficient of determination between per capita fat supply and dependent variables-overweight and obesity in two income groups after removing outliers

| **Correlation** | **Overweight** | | | **Obesity** | | |
| --- | --- | --- | --- | --- | --- | --- |
|  | R | p | R^2^ | r | p | R^2^ |
| Low | 0.49 | 0.02 | 0.24 | 0.67 | <0.001 | 0.45 |
| Upper middle | 0.36 | 0.09 | 0.13 | 0.43 | 0.04 | 0.18 |

r = Pearson’s correlation coefficient, p= Significance, R^2^=coefficient of determination

Low-income economies outliers= Yemen, Haiti; Upper-middle-income economies outliers= China
